# Processing binding data using an open-source workflow

**DOI:** 10.1186/s13321-021-00577-1

**Published:** 2021-12-11

**Authors:** Errol L. G. Samuel, Secondra L. Holmes, Damian W. Young

**Affiliations:** 1 Center for Drug Discovery, Baylor College of Medicine, One Baylor Plaza, Houston, TX 77030 USA; 2 Department of Pharmacology and Chemical Biology, Baylor College of Medicine, One Baylor Plaza, Houston, TX 77030 USA; 3 Department of Pathology and Immunology, Baylor College of Medicine, One Baylor Plaza, Houston, TX 77030 USA

**Keywords:** Thermal shift assay, Differential scanning fluorimetry, Fragment-based drug discovery, Fragment-based ligand discovery, KNIME, Education, Data analysis, Data processing, Workflows, Flow-based programming

## Abstract

The thermal shift assay (TSA)—also known as differential scanning fluorimetry (DSF), thermofluor, and T_m_ shift—is one of the most popular biophysical screening techniques used in fragment-based ligand discovery (FBLD) to detect protein–ligand interactions. By comparing the thermal stability of a target protein in the presence and absence of a ligand, potential binders can be identified. The technique is easy to set up, has low protein consumption, and can be run on most real-time polymerase chain reaction (PCR) instruments. While data analysis is straightforward in principle, it becomes cumbersome and time-consuming when the screens involve multiple 96- or 384-well plates. There are several approaches that aim to streamline this process, but most involve proprietary software, programming knowledge, or are designed for specific instrument output files. We therefore developed an analysis workflow implemented in the Konstanz Information Miner (KNIME), a free and open-source data analytics platform, which greatly streamlined our data processing timeline for 384-well plates. The implementation is code-free and freely available to the community for improvement and customization to accommodate a wide range of instrument input files and workflows.

**Supplementary Information:**

The online version contains supplementary material available at 10.1186/s13321-021-00577-1.

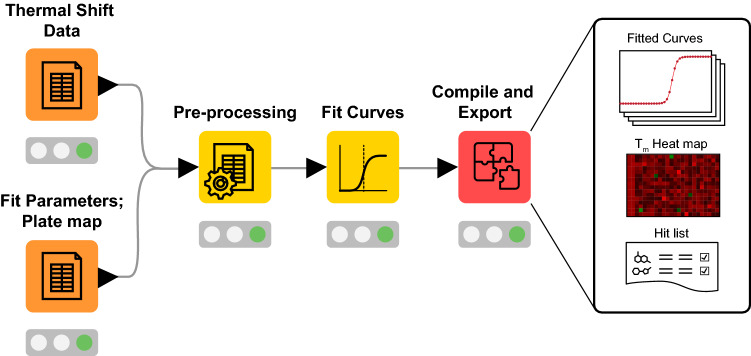

## Background

Modern small molecule protein modulators are developed using an array of drug discovery technologies such as fragment-based ligand discovery (FBLD), high-throughput screening (HTS), and DNA-encoded libraries (DELs). Library sizes and specific assays vary greatly, but irrespective of the preferred discovery technique, the ability to evaluate target engagement of every molecule in a screening library is essential to properly identifying novel binders and unlocking mechanistic details of newly developed drugs. Since screening libraries can be sizable collections of compounds, prodigious data sets are produced which require accurate and efficient methods of analysis to quickly identify hits. Academic labs are typically at a disadvantage when processing this data, as there are few methods that are both user-friendly and freely available. For users interested in such a method, this tutorial summarizes the widely popular thermal shift assay, discusses the existing approaches and workflows for thermal shift data processing, then outlines the development of a new, freely available, efficient cheminformatics workflow.

### The thermal shift assay

**Fig. 1:**
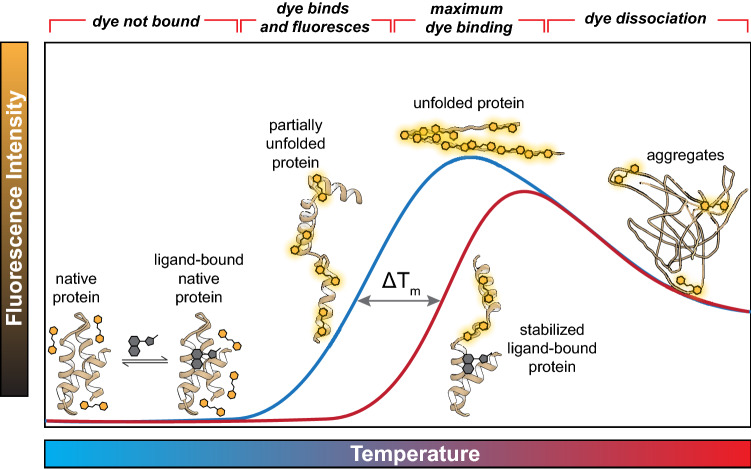
An example melting curve demonstrating protein denaturation with increasing temperature. The inflection point of the melting curve, which corresponds to the midpoint of the protein denaturation process, is the melting temperature (T_m_). Upon ligand binding, the stabilized protein exhibits a higher T_m_

The thermal shift assay (TSA) has become a mainstream technique to evaluate ligand binding owing to its ease of execution and high rate of throughput. Proteins fold in a manner that minimizes free energy. Hydrophobic residues pack into the interior of a structure and hydrophilic residues are exposed on the exterior [1]. The structures are stabilized by intramolecular forces such as electronic van der Waals attractions, dipole interactions, and hydrogen bonds [2]. As thermal energy increases, these intramolecular protein forces are disrupted, leading to protein structure destabilization, denaturation, and the exposure of interior hydrophobic sites. Thermal denaturation can be monitored by fluorescence detection with real-time PCR instruments using intrinsic tryptophan fluorescence, or environmentally sensitive dyes, i.e., dyes which have increased quantum yield upon binding hydrophobic environments [3]. A graph of fluorescence intensity vs. temperature yields a characteristic melting curve, and the temperature of heat denaturation (T_m_) is determined as the midpoint of the melting transition region. The T_m_ is influenced by sample conditions; bound ligands alter T_m_ values and therefore, experimentally significant ΔT_m_ observances are generally indicative of binding (Fig. 1) [4].

### Existing approaches and workflows for thermal shift data processing

At the bench, preparing for and performing a TSA protocol is straightforward. However, the subsequent data analysis, especially in a high-throughput format, is less intuitive and requires substantial preparation and manual manipulation. The two most employed methods to obtain melting temperatures from TSA data are the first derivative method and the curve fitting method—if the melting curve is not symmetrical, the T_m_ values determined by these two methods may differ. The first-derivative method determines this point by searching for the maximum of the first-derivative curve, or the minimum of the negative first-derivative curve. In the curve-fitting method, the melting curve is fit to a sigmoidal function, and the melting temperature can be determined by the inflection point parameter. Curve-fitting methods are known to be more robust and less sensitive to random noise than the first-derivative method [5, 6]. Moreover, a 5-parameter sigmoid provides a better fit particularly around the asymptotes compared to 4- or 3-parameter functions [7].

The data analysis process varies between laboratories, but often involves exporting the fluorescence data from the instrument, importing it to a separate software package to obtain melting temperatures, then exporting to another software package(s) for further analysis, hit determination, and visualization. The number of data transfers depends on the chosen form of T_m_ determination, available software, and the use of optional Microsoft^®^ Excel plug-ins. Some qPCR instrument manufacturers provide software for analysis of thermal shift data, to make this increasingly popular assay more user friendly, but these become less user friendly when analyzing data from multiple plates. Moreover, if the software use requires additional licensing, this introduces a potentially costly roadblock.

To meet this need, several groups have developed and shared tools for processing and analyzing thermal shift data, including curve fitting tools written in the MATLAB^®^ [7–9] and Python^®^ [10] programming languages, standalone all-purpose tools written in the Perl^®^ [6] and Java™ programming languages [11], and visualization tools based on Microsoft^®^ Excel [5]. DMAN, Meltdown, SimpleDSFviewer, and ThermoQ employ the first derivative method, while TSA-CRAFT and MTSA employ the Boltzmann fitting method, using 4-parameter and 5-parameter sigmoidal functions, respectively. Though these tools are designed to increase efficiency, in practice their usage becomes increasingly challenging due to the requirements of licensed software and proficiency in specific programming languages. Further barriers include the inability to customize the software if the lab qPCR instrument changes, or if new analysis features are desired. Screening hit identification is also optimal with structure comparisons and to the best of our knowledge, no existing tool integrates the chemical structures into the data analysis or visualization.

### Workflow/pipeline software. KNIME as an open-source data analysis tool

Workflow-based environments provide a common platform for multiple tools and have swiftly been adopted by the scientific community [12, 13]. The Konstanz Information Miner (KNIME) [14] is a free and open-source data analytics workflow platform which supports a wide range of functionality and has an active cheminformatics and bioinformatics community [15–25]. Its modular environment allows users to visually assemble and adapt the analysis flow from standardized building blocks called nodes, which are then connected through pipes carrying data or models. Nodes can be executed selectively and intermediate results checked via a graphical user interface. Metanodes and components can be used to encapsulate parts of a workflow and can even be re-used between workflows, allowing for the creation of complex but well-structured workflows. The capabilities of KNIME can be expanded by incorporating open-source third-party nodes including R integration, and open-source cheminformatics toolkits such as the RDKit, both of which are used in our workflow.

## Description of the KNIME workflow

**Fig. 2:**
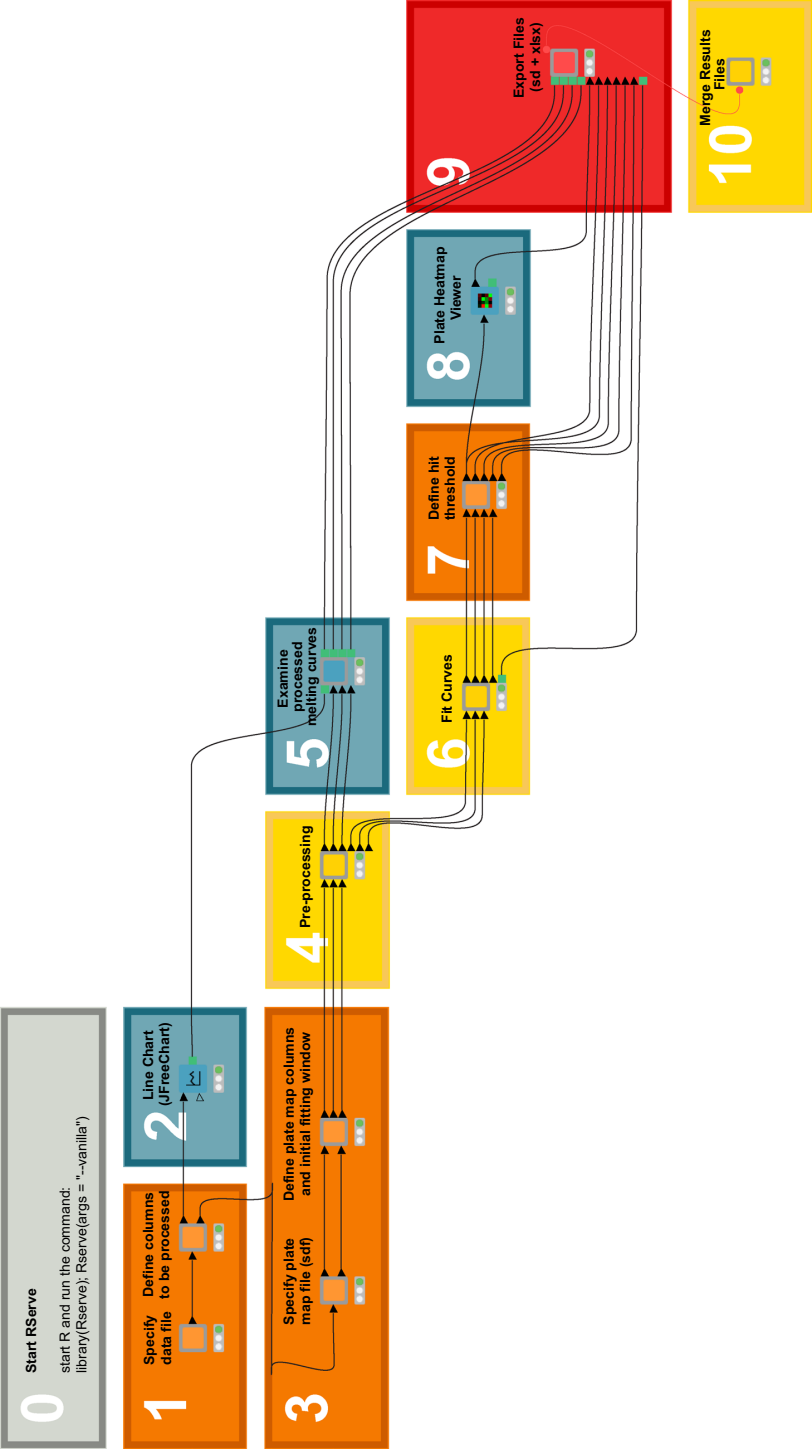
Thermal Shift Assay data analysis workflow in KNIME

While the workflow we describe here processes TSA data, the concepts and processes can be adapted to accommodate data from virtually any screening technique. Our workflow takes as inputs thermal shift assay data, a plate map containing chemical structures, and fitting parameters, and outputs melting temperature information for each compound. It has been designed as a sequence of 10 steps which guide the user through data analysis process and can be sequentially repeated for multiple plates (Fig. 2). Each step is color-coded in keeping with the KNIME node color scheme: orange is reserved for data input, yellow for data transformation, blue for exploration/visualization, and red for data output. This work was developed using KNIME version 4.4.0 and uses nodes from the KNIME Analytics Platform, open-source KNIME Extensions, and Community Extensions by RDKit, R, and the High-Throughput Technology Development Studio (TDS) of the Max Planck Institute of Molecular Cell Biology and Genetics (MPI-CBG). The RDKit extension is a collection of nodes that provides basic chemistry and cheminformatics functionality. The Interactive R Statistics Integration comprises nodes that interact with an external R™ installation [26] to write and execute R scripts. The high-content screening nodes provided by the MPI-CBG provide a powerful solution for mining screening data that is easy to use, but flexible enough to cope with various types of assays. These extensions should be downloaded once prior to first use. The KNIME workflow is freely available for download at https://github.com/loicsamuel/knime-tsa-analysis. Instructions for users explaining how to install and use the workflow are also provided at the same link and in Additional file 1.

### Step 0: R server initialization

The first step of the workflow is to start the R server by running R and executing the﻿ command:
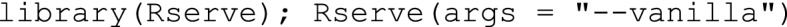


After command execution, the KNIME platform can communicate with the R server for executing nodes of the Interactive R Statistics Integration. Non-linear curve fitting of the melting curves is carried out using the Dose Response (R) node which uses the ‘drc’ R package [27] as a back end and ‘ggplot2’ for plotting the fit output. In the event that this workflow is used successively, step 0 is not required on subsequent runs.

### Step 1: input data

**Fig. 3:**
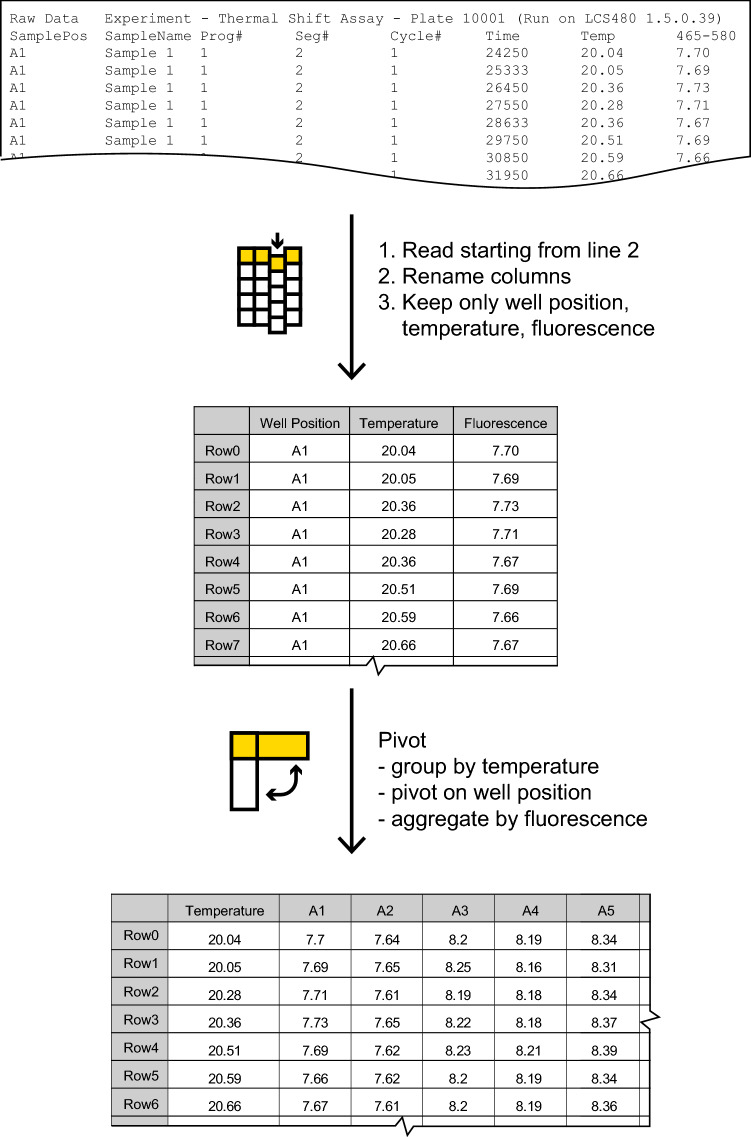
Reformatting a tab-separated text file containing thermal shift data exported from the Roche LightCycler^®^ 480 II for further processing in the workflow

The components in Step 1 reformat the raw output data from qPCR instruments into a table containing columns for well position, temperature, and fluorescence. These fields are the guiding requirement for workflow compatibility with a text file from any qPCR instrument, so users are asked to define these columns for the second component of this step. The data is then split into 2 outputs. The first is pivoted into a single temperature column and columns containing fluorescence values for each well at each temperature point, then transferred to step 2 for plotting, while the second output is transferred unchanged to step 3 for further processing (Fig. 3). We have successfully imported and processed the data file output from a Roche LightCycler^®^ 480 II instrument, but the step 1 component can be modified to accept and reformat any common file output. For example, we have also successfully imported a pre-processed Microsoft^®^ Excel output from the Applied Biosystems QuantStudio 7 Flex Real-Time PCR System.

### Step 2: examine melting curves

The user is encouraged to examine the melting curves to confirm that the majority are well-behaved, ideally with a flat initial pretransition region followed by a steep sigmoidal unfolding region, and finally an aggregation region, as shown in Fig. 1. The user should decide on a threshold for excluding wells which do not have sufficient fluorescence signal for reliable fitting and will therefore not yield sensible results. This step is also critical in the selection of an appropriate initial fitting window, as restricting the fitting algorithm to the transition region improves workflow performance.

### Step 3: experiment details

In step 3, the user is prompted to provide a plate map, in the form of an SD file, containing all library compounds and their locations (chemical structure, compound ID, plate ID, row, column, etc.), the initial fitting window, and a threshold for excluding wells which lack a suitable fluorescence signal. As an alternative to the SD format, a workflow is provided which accommodates a plate map of compound SMILES in CSV format. Example data, along with plate maps and KNIME workflows are provided as Additional files 2, 3, 4, 5, 6 and 7. The plate number, row, and column data contained in the plate map are used to associate the chemical structures and compound IDs with the temperature and fluorescence data. The joined data is then passed on to subsequent nodes which remove data outside of the fitting window and plot the resulting curves.

### Step 4: flag problematic wells

First, the range of fluorescence values in each well is calculated and compared to the threshold specified in step four. Wells that do not meet the fluorescence signal threshold are flagged and separated for plotting and review in step 5. The user should examine these melting curves to determine whether they were correctly flagged. The fluorescence range threshold should be adjusted until the filter captures, as much as possible, only the low-signal curves. Melting curves that meet or exceed the threshold are then analyzed for the presence of a downward slope or high initial fluorescence, i.e., whether the global maximum fluorescence occurs at a lower temperature than the global minimum fluorescence. After this, the curves are analyzed for the presence of gaps, jumps, and spikes. By default, these are defined as regions where Δ*f* (the change in fluorescence between two successive data points) is more than 25 times the mean Δ*f* for that well. This criterion can be modified to suit the data being processed—noisy data would require a higher threshold. We suggest starting with a lower value then increasing it if necessary.

### Step 5: review pre-processing results

**Fig. 4:**
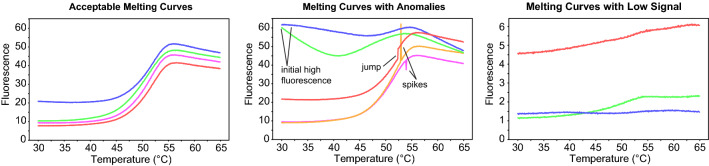
Examples of acceptable melting curves, melting curves which have been flagged as containing anomalies, and melting curves with insufficient signal for curve fitting

Here, the user can review the processed melting curves to confirm that the shapes are sigmoidal with well-behaved minima and maxima, and a sigmoidal transition region that is completely inside the fitting window. Importantly, the user is also advised to confirm whether the flagged spectra are indeed problematic, and to adjust the thresholds and the fitting window of step 3 to avoid anomalies while capturing as much real data as possible. Figure 4 illustrates several types of melting curves which are flagged by this component. The well selection feature in Step 1 allows for the isolation of problematic individual wells if desired, and a T_m_ can be calculated on these by adjusting the initial window for a more custom fit.

### Step 6: non-linear regression

**Fig. 5:**
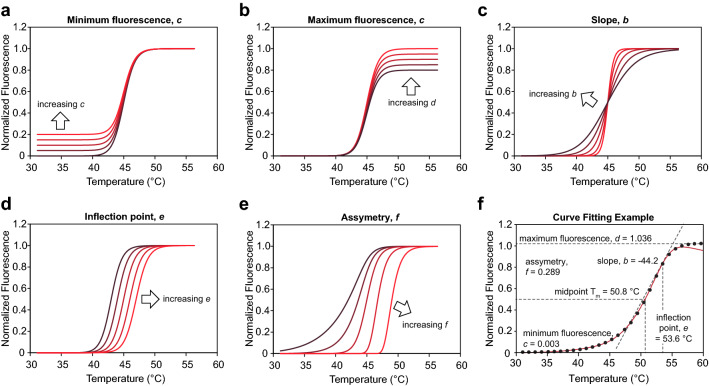
Illustration of the parameters used in 5-parameter log-logistic fitting. **a**–**e** Effect of increasing minimum, maximum, slope, inflection point, and asymmetry values, respectively. **f** melting curve data (red line) fit to a 5-paremeter log-logistic function (black dots), and the resulting fit parameters

Inside this component is a loop, which for each well,

1. Applies a 21-point center simple moving average for smoothing of the fluorescence values
2. Normalizes fluorescence values between 0 and 1
3. Determines the temperature value at which the minimum fluorescence value occurs
4. Discards fluorescence data which occur at lower temperatures than the value determined in (3)
5. Determines the temperature value at which the maximum fluorescence value occurs
6. Discards fluorescence data which occur at higher temperatures than the value determined in (5)
7. Fits the fluorescence data from each well to a 5-parameter log-logistic model
  $$f\left(x\right)=c+\frac{d-c}{{\left[1+\mathrm{exp}\left(b\left(\mathrm{ln}\left(x\right)-\mathrm{ln}\left(\mathrm{e}\right)\right)\right)\right]}^{f}}$$
  where *b* = slope, *c* = minimum fluorescence value, *d* = maximum fluorescence value, *e* = inflection point, and *f* = asymmetry factor, i.e. the difference in the rates of approach from the inflection point to the lower and the upper asymptotes (Fig. 5). If the melting curve is symmetrical then *f* = 1 and the equation becomes a 4-parameter log-logistic.
8. The inflection point of an asymmetric sigmoidal curve does not coincide with its midpoint. The midpoint T_m_ is calculated using the fit parameters according to the equation
  $$midpoint {T}_{m}=\frac{e}{\mathrm{exp}\left[-\frac{1}{b}\mathrm{ln}\left({2}^\frac{1}{f}-1\right)\right]}$$

The component returns the melting temperature, fit parameters with error estimates, and the fitted curves.

### Step 7: define hit threshold and compile results

Once fitting is complete, the user can define the hit threshold for hit selection by double clicking on the component in Step 7. By default, this is set to 3 times the standard deviation of the melting temperatures of the DMSO-treated wells. The component splits the data into two branches for further manipulation. The first branch groups the logistic fit results by molecule ID number and calculates the average melting temperature and standard deviation, thereby providing a summary of replicates if present. The melting temperatures are then compared to the previously defined hit threshold and hits are tagged as such. The second branch creates a heatmap of the plate, colored by melting temperature.

### Step 8: review melting temperature heatmap

**Fig. 6:**
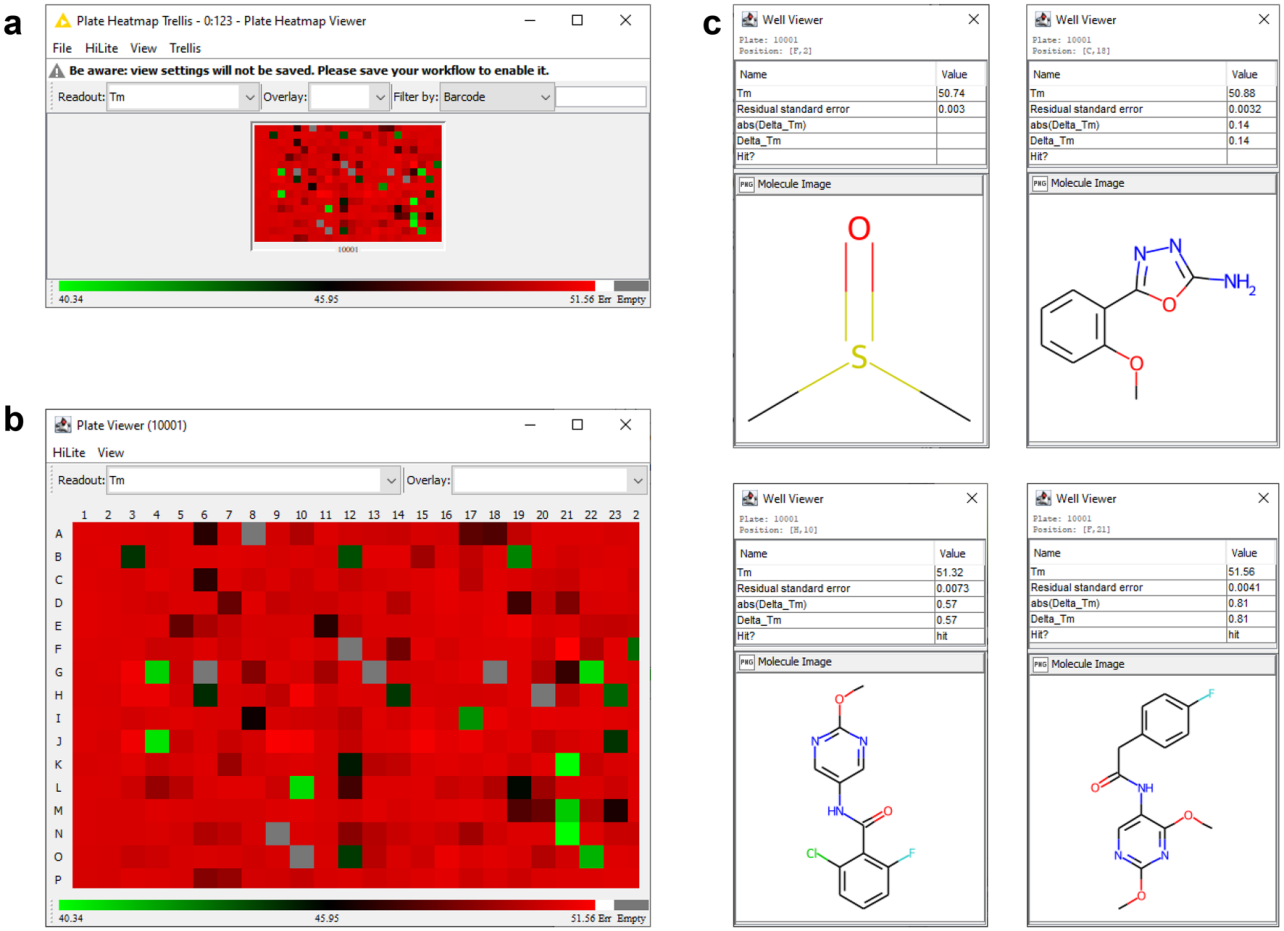
Heatmap of melting temperatures. **a** Trellis view showing all plates in the experiment. **b** Detailed plate view. **c** Detailed well views

At this stage, the user is encouraged to inspect the array of melting temperatures using the Plate Heatmap Viewer node. This node provides access to three levels of data with corresponding levels of detail: screen → plate → well. The screen view (Fig. 6a) is the default view, a trellis containing each plate in the experiment. This view is invaluable for visualizing row and column artifacts and for comparing plate performance across a screening campaign. For this workflow there is only one plate. The *View* menu provides the ability to change the colormap, while the trellis toolbar allows the user to change the readout as well as overlaid information, such as a hit tag. The second data level is the plate view (Fig. 6b) which is accessed by right-clicking on a plate in the heatmap trellis. There are identical controls as in the screen view, and individual wells can be inspected by hovering over them with the mouse. Right-clicking on a well provides access to the third and final data level, the well view (Fig. 6c). This view provides a detailed view of the wells along with information such as well position, compound ID, and chemical structure. The user is advised to note any patterns that may indicate the presence of problematic wells, e.g., lack of consistency in DMSO control wells, or systematic errors such as the presence of edge effects, outlier rows/columns, or unexpected patterns for ostensibly randomly plated compounds.

### Step 9: output

The workflow produces two files, located in the same folder as the initial thermal shift data file and sharing the same name. The first is an SD file containing all the columns of the initial plate map file along with melting temperature, residual standard error of the fit, ΔT_m_, |ΔT_m_| and hit status. Alternately, a second workflow is provided which outputs a CSV file with compound SMILES instead of an SD file. The second output is an XLSX document containing various sheets:

- *Plate Heatmap:* this includes a heatmap of the melting temperatures across the plate
- *Fit Results with hits annotated:* a list of all fitted compounds including (i) an image of the molecule, (ii) molecule ID number, (iii) well position, (iv) plate row, (v) plate column, (vi) plate ID number, (vii) melting temperature, (ix) residual standard error, (x) ΔT_m_, (xi) |ΔT_m_|, (xii) hit status
- *DMSO mean and SD:* this includes (i) molecule ID, (ii) control T_m_, (iii) standard deviation of control T_m_, (iv) hit threshold as 3 × standard deviation of control T_m_, (v) hit threshold as control T_m_ + 3 × standard deviation of control T_m_
- *Fitted Melting Curves:* this includes the well position and a graph of the sigmoidal fit for each fitted well
- *Melting Curves:* this includes the graphs showing the overlaid raw melting curves, cropped melting curves, accepted melting curves, melting curves with anomalies, melting curves with low signal, and processed melting curves used for fitting.

### Step 10: data consolidation

Data files loaded into the KNIME workflow during step 1 will typically consist of TSA raw fluorescence data from a single plate run of the qPCR instrument, but screening libraries are typically large enough to span multiple plates. The next steps in the screening project are to compile and compare all available data and it is inconvenient to have that data spread across the multiple files that will be produced from multiple runs of the workflow. This last (optional) workflow component merges the results files produced during step 9. When executed, the component in step 10 reads all SD (or CSV) files saved in the “Results” folder and combines the data into a single table which is then is saved as both XLSX and SD (or CSV) files.

## Conclusion

**Fig. 7:**
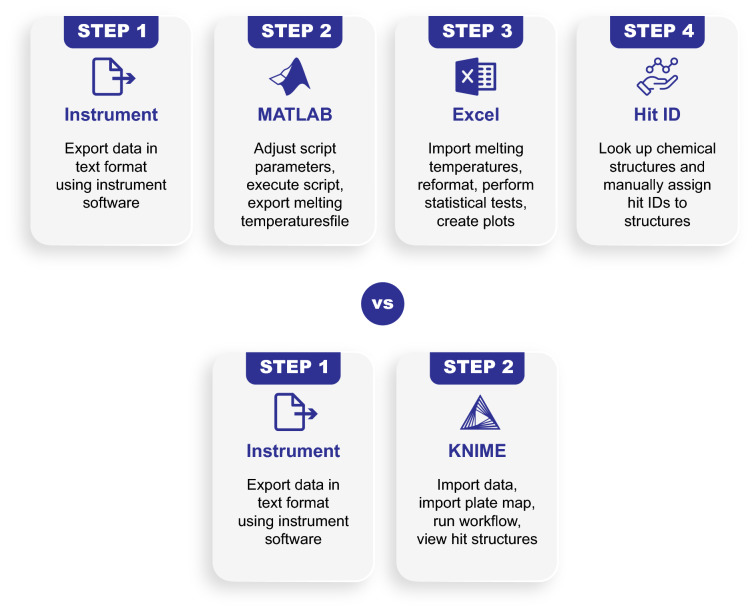
Comparison of TSA data processing approaches. **a** our previous process including curve fitting in MATLAB, collation and further analysis in Microsoft Excel, and finally, chemical structure review in a software package such as DataWarrior. **b** One-step KNIME workflow

The KNIME workflow developed and described in this paper provides an efficient and user-friendly method for processing both small and large TSA datasets. It provides significant savings both in time and financial resources (Fig. 7), and we hope it will serve as a valuable tool for researchers, particularly in academic institutions with smaller budgets and lower data analytics and programming expertise.

This type of workflow is accessible and can be used as a model for analysis of large datasets from virtually any other screening technique. We encourage the community to apply this tool in their scientific work and to improve it based on their knowledge, experience, and specific data processing needs.

## Supplementary Information

**Additional file 1**. Workflow Instructions.

**Additional file 2**. Thermal Shift Data (Plate 10001).

**Additional file 3**. Thermal Shift Data (Plate 10002).

**Additional file 4**. Plate Map (SD format).

**Additional file 5**. Plate Map (CSV format).

**Additional file 6**. KNIME Workflow (SD format).

**Additional file 7**. KNIME Workflow (CSV format).

## Data Availability

The KNIME workflow and accompanying data are included within the article (and its additional files) and on GitHub (https://github.com/loicsamuel/knime-tsa-analysis).

## Abbreviations

CSV: Comma separated variable
DEL: DNA-encoded library
DNA: Deoxyribonucleic acid
DSF: Differential scanning fluorimetry
FBLD: Fragment-based ligand discovery
KNIME: Konstanz information miner
PCR: Polymerase chain reaction
PROTAC: Proteolysis targeting chimera
SDF: Structure data file
TSA: Thermal shift assay
T_m_: Melting temperature
