# Supplementary material for: Processing binding data using an open-source workflow
Source: J Cheminform. 2021 Dec 11;13:99. doi: 10.1186/s13321-021-00577-1 (PMC8666039; doi:10.1186/s13321-021-00577-1)
Supplement: Supplementary file 1 — Additional file 1. Workflow Instructions. [file 13321_2021_577_MOESM1_ESM.docx]

# An Open-Source Workflow for Processing Binding Data

# Installation

## Install and configure R

Instructions for installing R and required packages can be found in the [official KNIME documentation](https://docs.knime.com/2019-12/r_installation_guide/index.html) and the [Rserve github page](https://github.com/knime-mpicbg/knime-scripting/wiki/R-server-for-knime#Install_packages_required_for_R_templates_in_Knime). For this workflow, the packages ‘drc’ and ‘ggplot2’ are required.

## Install KNIME

Install the latest version of KNIME using the appropriate installer at <https://www.knime.com/downloads/download-knime>

## Install the workflow

1. Download the KNIME workflow from https://github.com/loicsamuel/knime-tsa-analysis
2. Open KNIME
3. Go to File →*Import KNIME Workflow*
4. Click “*Select File”* then “*Browse…”* and select the workflow .knwf file
5. Click *“Finish”*
6. The workflow will appear in the KNIME Explorer menu. Double click on the workflow to open it.
7. A message may appear asking you to install missing extensions which are used in the workflow. Click “*Yes”* and follow the on-screen instructions.


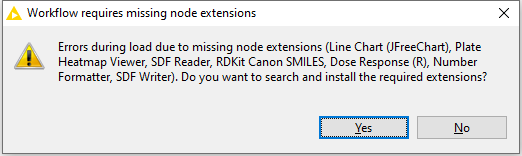


# Using the Workflow

Gather input files.

## Step 0. Start RServe

Start R, then execute the command library(Rserve); Rserve(args = "--vanilla")

## Step 1a. Load thermal shift data

1. A red lit traffic light beneath the *“Specify data file”* node indicates that it needs to be configured. Double click on the component to open a dialog box for node set up.


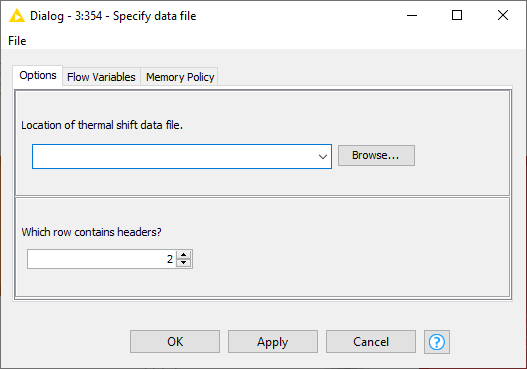


1. Click “Browse...”, select the input file, then click *“OK”*
2. Enter the number of the row which contains column headers then click *“OK”*
3. Execute the component in one of three ways:
   1. Select the component and press F7
   2. Select the component and click
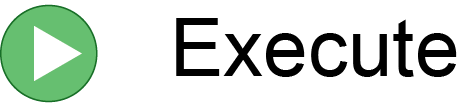
 on the main toolbar
   3. Right click on the component and then select
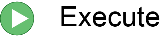
 in the context menu
4. When executed successfully, the traffic light symbol beneath the component will change from red to green.

## Step 1b. Load thermal shift data

1. Double click on the node labelled “Define columns to be processed”


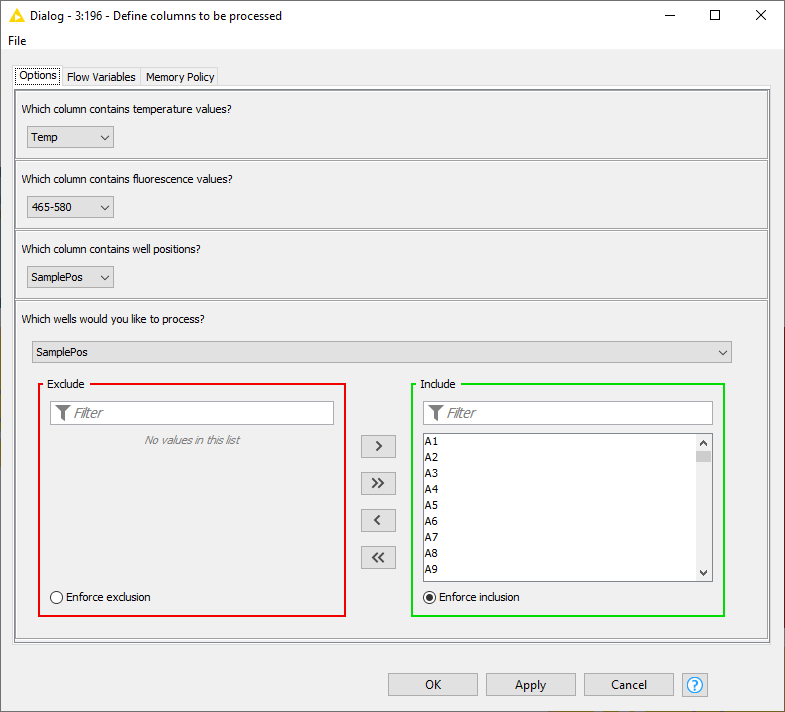


1. Specify the names of the column that contains temperature values, fluorescence values and well positions
2. Ensure that all wells to be processed appear in the “*Include”* column, then click *“OK”*.
3. Execute the component.

## Step 2. Visualize melting curves

1. Execute the Line Chart node
2. View the output by selecting the node and clicking the
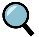
 icon on the main toolbar.

An example is shown below, derived from data that will serve as a guiding illustration throughout this instructional manual.


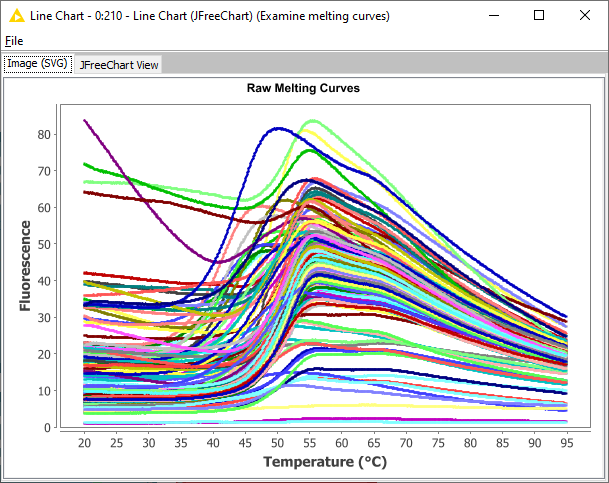


1. Make a note of the temperature region where the melting transition occurs. Also note the presence of any potentially problematic melting curves, such as curves with little to no signal, or curves with spikes, gaps, or jumps.

## Step 3a. Specify Plate Map File

1. Double click to configure the component


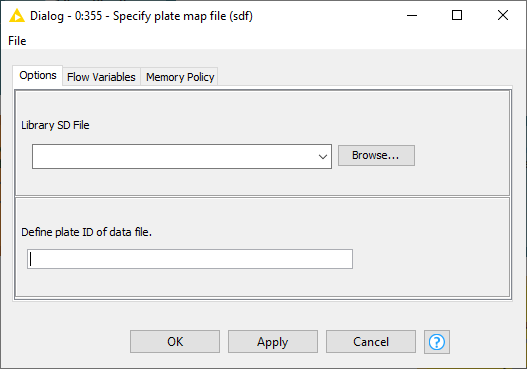


1. Define the path to the library SD file
2. Specify the ID of the plate being screened
3. Execute the component

**Step 3b. Define Processing Parameters**

1. Double click to configure the component


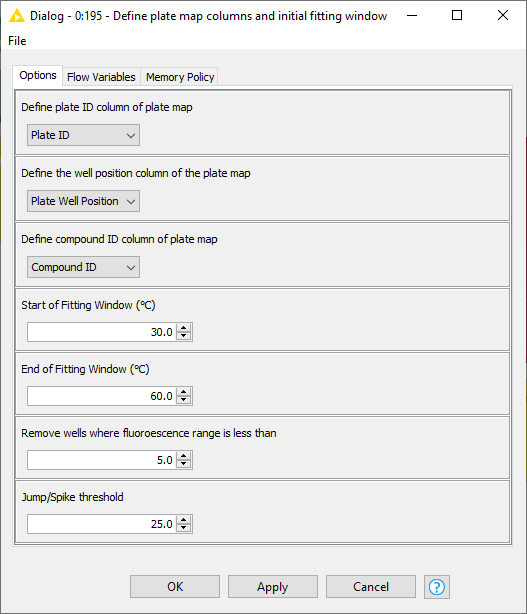


1. Define the name of the column containing the plate ID
2. Define the name of the column containing the well positions
3. Define the name of the column containing the compound IDs
4. Define the start and end of the fitting window

For the demo data, a range of 30 to 60 °C is suggested as the initial fitting window, based on the Raw Melting Curves chart.

1. Define the low signal threshold

The demo fluorescence signal threshold is set to 5 fluorescence units. This will filter out curves which show no transition and are senseless to try and fit.

1. Define the jump/gap/spike threshold

The demo data contains melting curves which contain jumps and spikes. A threshold of 25 is sufficient to flag these curves. A higher threshold would flag fewer melting curves, while a lower threshold would flag more.

1. Click *“OK”*
2. Execute the component

## Step 4. Pre-Process Melting Curves

Execute the component. There is no configuration required.

## Step 5. Examine Processed Melting Curves

As with the line chart in Step 2, view the component output by clicking the
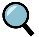
 icon on the main toolbar of the node

1. The first plot displays the melting curves which passed the basic quality tests. Confirm that the fitting window is wide enough to include the steep sigmoidal unfolding region for (ideally) all the curves
2. The second plot displays melting curves that exhibit high initial fluorescence, as well as curves which may exhibit jumps, gaps, or spikes. Confirm the presence or absence of melting curves. If curves are plotted which do not have any visible anomalies, increase the jump/gap/spike threshold, as the data may be noisy.
3. The third plot displays melting curves that have a low signal. Confirm that the signal threshold was sufficiently high to capture offending curves, but sufficiently low to avoid false positives.
4. If parameter refinement is required, return to step 3.


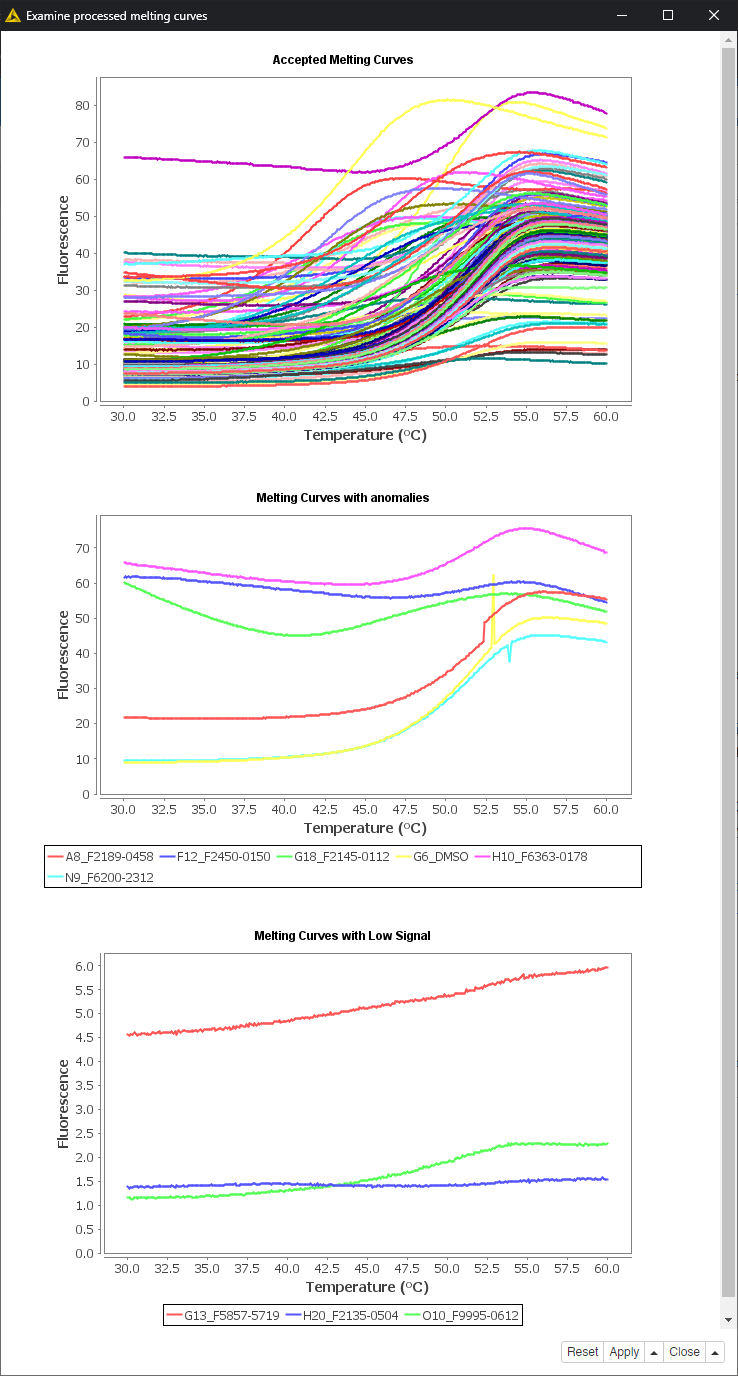


## Step 6. Fit Curves

There is no configuration required to execute the component of step 6. Depending on the number of wells being processed, the number of data points per curve, and the specifications of your computer, execution of this step may take up to 30 minutes.

## Step 7. Define hit threshold and compile results

1. Double click on the component


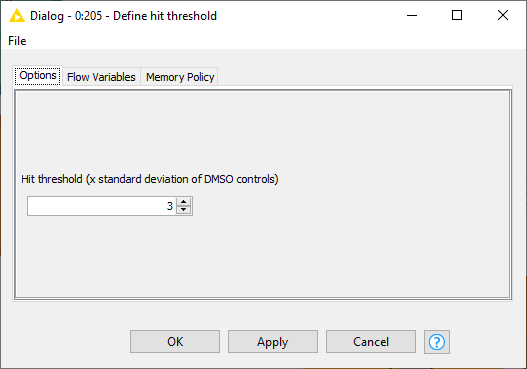


1. Define the hit threshold, then click *“OK”*
2. Execute the component

## Step 8. View Heatmap with Melting Temperature Results

1. Execute the node
2. View the heatmap output by clicking the
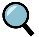
 icon on the main toolbar. A trellis view should appear


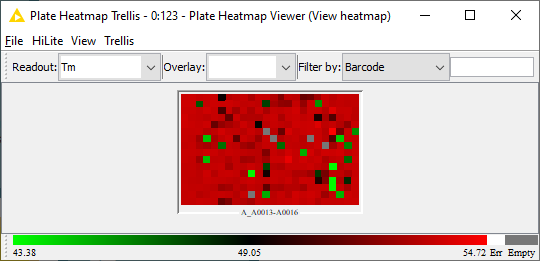


1. Right-clicking on the heatmap will open a more detailed view that gives information about each well including structure information.


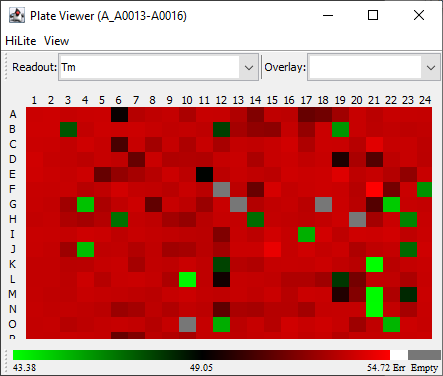


1. Selecting *“Hit?”* from the Overlay drop down menu will highlight wells with hits


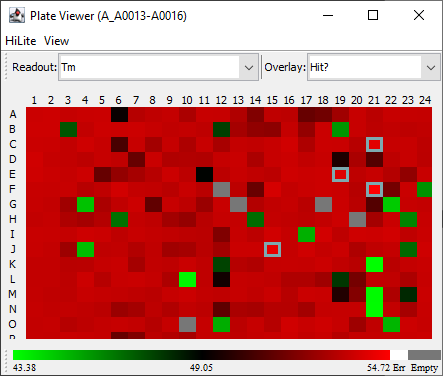


1. Right-clicking on an individual well will open another window with details about that well
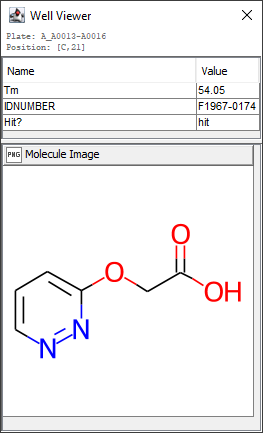


## Step 9. Export Results

Executing this component will create two files. The first is an SD file containing the fitted compounds, their melting temperatures, and their status as a hit. The second is a Microsoft Excel® file containing multiple sheets of data showing:

- 1. the melting temperature mean and standard deviation of DMSO-treated wells
  2. fit results with the plots of hits tagged as such
  3. the plate heatmap
  4. each fitted melting curve
  5. temperature vs fluorescence data for curves with anomalies
  6. temperature vs fluorescence data for curves with low signal
  7. the plot of original melting curves
  8. the plot of processed melting curves
  9. the plot of melting curves which contain anomalies
  10. the plot of melting curves exhibiting low signal

These files will bear the same name as the original data file and will be located in the “Results” folder. The component will fail to execute if either output file already exists.

## Step 10. Consolidate Results

This component will merge data produced from previous runs of steps 1-9. An SD file and a Microsoft Excel® file named “Merged Results” will be saved containing all information within the individual SD results files. It will be located inside a new “Merge” folder nested in the “Results” folder and is overwritten upon every execution. To protect data, please move and rename this file after completing the final merge of each screen.
